# Physicochemical Biology: Conquered Boundaries and New Horizons 

**Published:** 2012

**Authors:** D.G. Knorre

**Affiliations:** Institute of Chemical Biology and Fundamental Medicine, Siberian Branch, Russian Academy of Sciences, prosp. Akademika Lavrent’eva, 8, 630090, Novosibirsk, Russia

**Keywords:** DNA structure, enzyme active sites, unstructured proteins, dynamics of biochemical processes, single molecule studies of enzymatic processes and biopolymer tertiary structure formation

## Abstract

In this paper, we shall consider the main evolutionary stages that occurred within the field of physicochemical biology during the 20th century, following the determination of the tertiary structure of DNA by Watson and Crick and the subsequent successes in the X-ray structural analysis of biopolymers. The authors’ ideas on the pre-emptive problems and the methods used in physicochemical biology in the 21st century are also presented, including an investigation of the dynamics of biochemical processes, studies of the functions of unstructured proteins, as well as single-molecule investigations of enzymatic processes and of biopolymer tertiary structure formation.

The second half of the 20th century represented a period of tremendous achievement for mankind, witnessing an increase in the understanding of the essence of natural phenomena and heralding significant breakthroughs with regard to mankind’s technical abilities. Progress achieved in the use of nuclear energy has enabled us to cover the earth with a network of nuclear power stations, while the appearance of powerful jet engines has opened the doors to space voyage; a process which began with Gagarin’s flight, continued with the landing on the moon, and is now moving towards a flight to Mars. Advancements in electronics and materials science have enabled us to build computers that can perform trillions of computations per second and have facilitated the creation of devices smaller than a matchbox that are capable of storing gigabytes of information, as well as systems for ultrahigh-speed information transfer. These factors have resulted in the emergence of one of mankind’s most impressive technical marvels: the Internet.  

A less obvious, but no less significant, achievement was accomplished in our understanding of the chemical and physical foundation of the functioning of living organisms. It is fair to say that the introduction of physical and chemical approaches into the field of life sciences was a gradual process; this progress made a significant leap forward after the publication of the famous paper by James Watson and Francis Crick in 1953, which presented a three-dimensional structure of deoxyribonucleic acid (DNA) [1]. 

A plethora of mysteries exist within nature. Amongst these is the question of how an unthinkably huge amount of information is transferred accurately from one cell produced by the fertilization of an egg cell to an adult organism. Until 1944, the nature of the carrier of hereditary information remained unknown. Although Miescher had discovered nucleic acids in 1869, the prevailing view was that a protein played the role of such a carrier; indeed, it was not until 1944 that Avery demonstrated that DNA was the actual carrier of hereditary information [2]. 

It was evident that such a carrier had to have three main functions. First of all, it was necessary for the carrier to possess a huge storagecapacity with which to store information relating to the manifold properties of a living organism, including its structure and the functions inherent to specific species and even individuals. Secondly, such a carrier should possess a mechanism for the realization of the information in to the definite structures of a living organism and its numerous functions (in order to express this information). Thirdly, the most important requirement was the existence of a mechanism for transferring the information to subsequent generations. 

The main claim of DNA to be such a carrier is rooted in its chemical structure. DNA is a linear polymer that is comprised of four different monomers, i.e.nucleotides. Each monomer consists of three fragments: a carbohydrate residue (deoxyribose) bound to an orthophosphoric residue and one of the four heterocyclic residues: adenine, guanine, thymine, and cytosine. 

**Figure:**
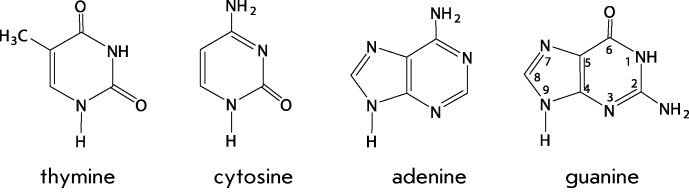


The nucleotides are bound by phosphodiester bonds between deoxyribose and phosphoric acid residues ( *Fig. 1* ).

**Fig. 1:**
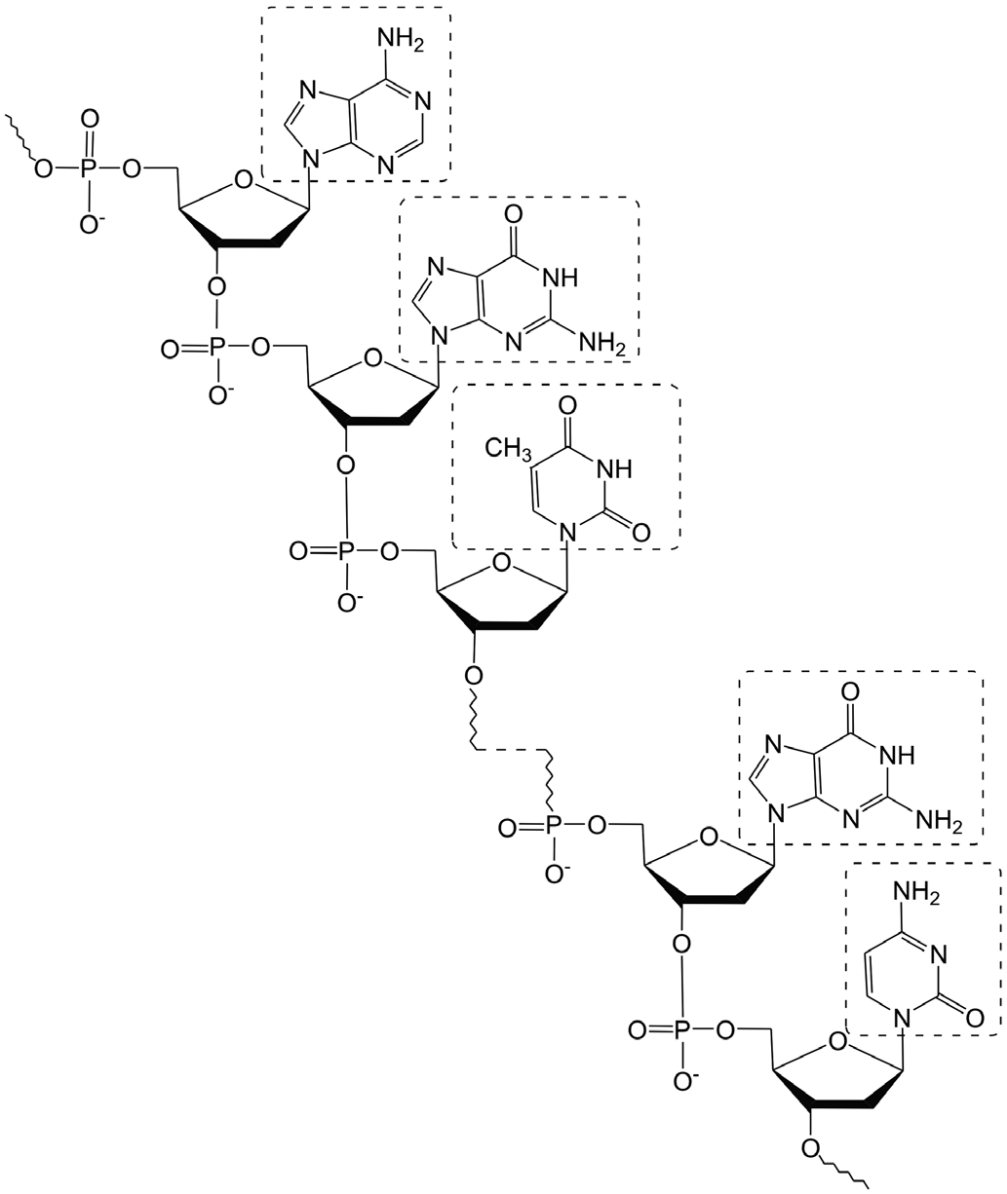
Fragment of the structure of a DNA molecule.

Such a structural principle enables the existence of an innumerable quantity of various polymeric structures differing in the set and sequences of nucleotides. For a polymer built from *n* monomeric units, the number of combinations amounts to 4 ^n^ = 10 ^0.6n^ . Even for a very short polymer of 200 monomeric units, this amount (10 ^120^ ) exceeds the number of atoms in the observable part of the universe, which is estimated at (10 ^80^ ). In fact, the DNA for even the simplest organisms is comprised of thousands, even millions, of nucleotides. 

These calculations imply that most imaginable nucleotide sequences could not, in principle, appear in the universe and are subjected to natural selection. This means that the appearance or generation of organisms more fascinating than those that exist at the present time can not be excluded. 

However, neither the light shed upon the role of the DNA as an information carrier, nor the huge information capacity of the DNA molecule, has enabled us to solve one of the most intriguing puzzles of this area of science: i.e., how this vast amount of information is transferred from one generation to the other. It has been established that the answer to this puzzle is rooted in the spatial structure of the DNA. According to the model proposed by Watson and Crick,which has been fully confirmed by numerous subsequent experimental studies, DNA is a construct of two polynucleotide chains which are bound together by hydrogen bonds. Within the structure proposed, adenine may interact only with thymine; and guanine with cytosine. Such sequences are considered complementary. The presence of such a correspondence means that any nucleotide sequence in one chain unambiguously corresponds to one nucleotide sequence in another chain, hereby following the mechanism of information transfer from maternal to two daughter cells at cell division. According to this mechanism, two polynucleotide chains separate prior to cell division, each of them governing the formation (synthesis) of a new complementary chain, thus double-stranded structures are formed that are identical to the DNA of the maternal cell. The existence of such a process was confirmed by Meselson and Stahl [3] soon after the appearance of Watson-Crick’s work. These authors prepared *Escherichia coli* cells grown on a medium containing ^15^ NH _4_ Cl as a single source of nitrogen. Thereafter, the cells were permitted to grow for several generations in a medium with the usual nitrogen isotope. In all subsequent cell generations, the presence of heavy DNA with the same amount of the heavy nitrogen isotope was observed; indicating that the ^15^ N-DNA formed in the first step of the experiment remained intact and was simply transferred to one of the daughter cells during each subsequent division of the daughter cells. 

The defining feature of Watson-Crick’s work was the fact that the structure of a biologically significant macromolecule was derived using the established geometrical parameters of definite chemical bonds; therefore, the elucidation of the biological phenomenon that begins with the physicochemical parameters of the molecule responsible for the phenomenon was achieved. Consequently, this work can be considered as having heralded the birth of physicochemical biology. 

Currently, physicochemical biology includes several scientific disciplines: biological chemistry, biophysics, bioorganic chemistry, and molecular biology. It can reasonably be argued that the traditional separation of these disciplines is not entirely appropriate. For example, molecular biology, according to Wikipedia, is defined as a science that deals with the molecular backgrounds of biological activity; however, biological chemistry has focussed for a considerable period of time upon molecular concepts which describe the most essential biochemical processes as being conversions of molecules with a commonly known chemical structure and has considered the catalysts of these processes to be individual compounds, i.e. as molecules. Therefore, the entire concept of modern biological chemistry refers to molecular science and could therefore reasonably lay claim to the appellation “molecular biology”. For the remainder of this paper the term physicochemical biology will be used to refer to the science that studies biological phenomena on the basis of the physicochemical properties of separate atoms and chemical bonds. 

The work of Watson and Crick inspired vigorous efforts which eventually resulted in the identification of the primary biochemical mechanisms that ensure the transfer and expression of genetic information. The concept of the matrix synthesis of biopolymers was the central element of these mechanisms; according to this, each step of elongation of a new biopolymer molecule is not only catalyzed by a specific enzyme, but is also checked by a special nucleic acid, indicating which monomer should be bound to the growing polymer chain at a given stage. These mechanisms are described in all contemporary international and Russian biological chemistry textbooks and manuals, such as [4, 5]. 

The discovery of the enzymes that catalyze the synthesis of complementary DNA molecules has led to the elaboration of the polymerase chain reaction (PCR) [6], which has found application in medical diagnosis, forensic science, and archaeology. 

The elucidation of the mechanisms of DNA expression and the achievement of chemists in the synthesis of oligonucleotides of a desired sequence has led to the appearance of genetic engineering [7]. It has become possible to carve out definite genes, to modify them and then to subsequently insert them into the proper region of the genome, thus performing site-directed mutagenesis [8]. 

The greatest scientific effort in the field of physicochemical biology was launched in 1990 under the name “Human Genome,”a program aimed at the mapping of the complete nucleotide sequence (sequencing) of human DNA [9]. As early as in 2001, Venter and 272 co-authors had published a complete nucleotide sequence of the human genome [10]. The methods elaborated within the framework of the program and those that are still being improved have opened the doors to the obtaining of genetic maps for any individual; as well as for the obtaining of genome structures for all the animals, plants and microorganisms on Earth. Consequently, irrespective of the striking success that has been achieved in the elaboration of high-speed efficient sequencing methods, scientists dealing with molecular biology have had enough work on their plate to last for several decades. 

The entire breadth of ground that physicochemical biology has covered, from Watson and Crick’s effort to the determination of the structure of the human genome, can be viewed as an incremental effort with clearly formulated tasks and with the purpose of investigating and designing new, innovative methods. During that journey, new and unexpected advances were made along the way; among such advances is the discovery of ribozymes by Thomas Cech [11] and Sidney Altmann[12],as well as the discovery of small interfering RNAs [13]. 

The appearance of new physicochemical peculiarities for living matter in the future is an eventuality which cannot be excluded *a priori* . The role of a significant portion of the human genome remains unclear, since only 1.5% of it is responsible for the 23,000 genes coding human proteins. A significant portion of the genome determines the synthesis of various non-coding RNAs: transfer and ribosomal RNAs, introns. However, this does not account for the remaining 98.5% of the genome,and thus a significant portion is considered junk DNA. Establishing the role of this DNA is one of the most challenging problems in the field of physicochemical biology. The functional importance of the extracellular nucleic acids present in appreciable amounts in the blood plasma still remains unclear [14]. 

Among the main achievements in physicochemical biology in the past century, it would be short-sighted not to mention the great progress achieved through X-ray crystallography and the NMR study of proteins in the understanding of the mechanisms of biological catalysis. A large body of material has been accumulated relating to the atomic structure of an enzyme’s active sites and their complexes with specific ligands, which has enabled the formulation of reasonable hypotheses pertaining to the mechanisms of recognition and catalytic conversions. For a perspective on the degree of information obtained on the nature of an enzyme’s active sites, a scheme of the arrangement of the reaction intermediate phenylalanine adenylate in the active site of the phenylalanine-tRNA-synthetase is presented in *Fig. 2,* with the bonds formed by enzyme active site groups, including the water molecules participating in the interaction [15]. 

However, this author believes that the focus of physiochemical biology in the 21 ^st^ century should shift to other matters. Several aspects which require primordial development both at the theoretical and experimental levels should receive more attention. First and foremost, the role of molecular dynamics requires significantly more attention. 

**Fig. 2:**
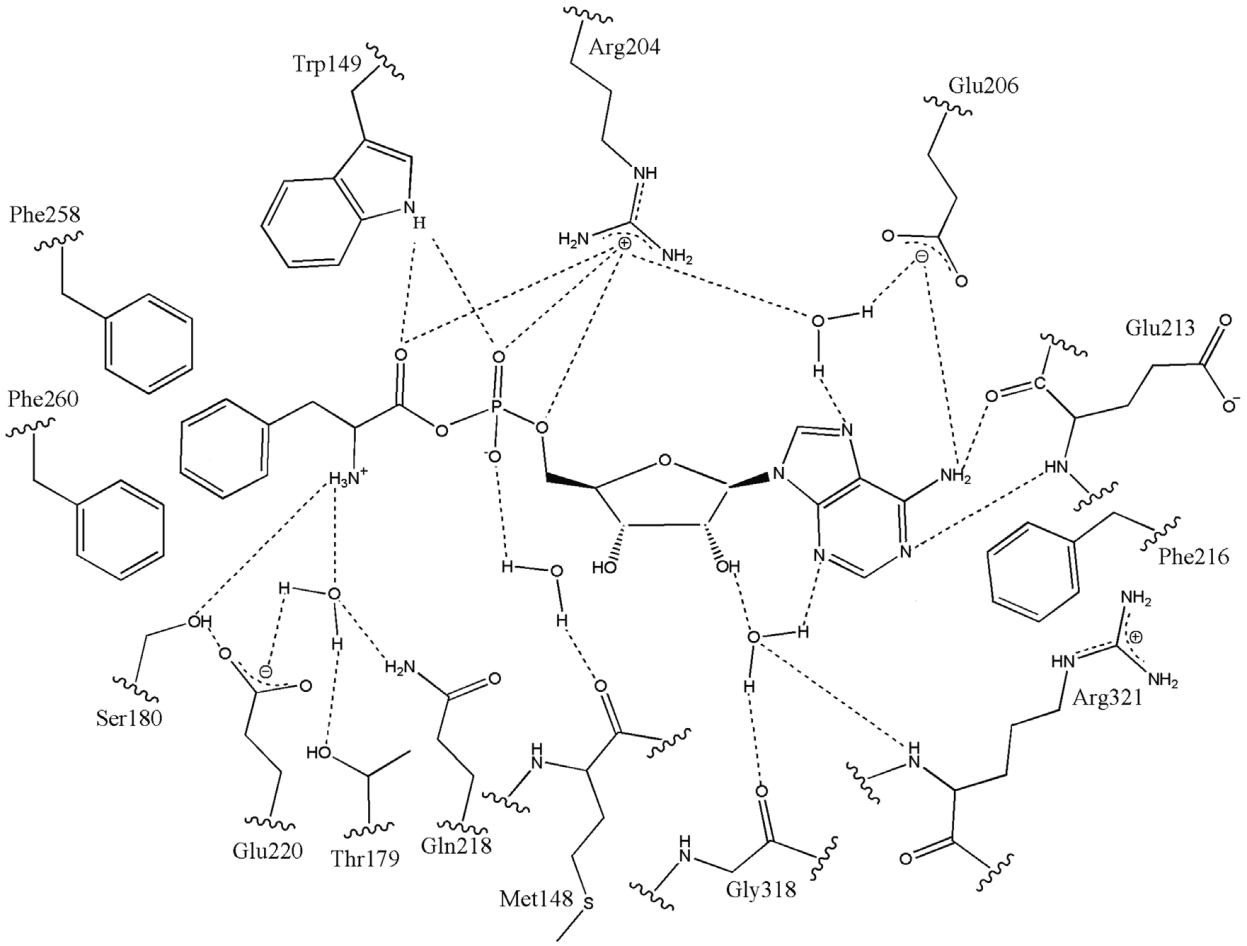
Structure of the active site of phenylalanine-tRNA-synthetase in complex with the intermediate (phenylalanine adenylate). Dots indicate hydrogen bonds between the intermediate atoms and the enzyme with binding water molecules.

Certainly, the dynamic character of the functioning of the biopolymer did not come as an unexpected revelation. However, organic chemistry, including bioorganic chemistry, has dealt predominantly with structures that are, in essence, static. Dynamic events have been considered as a transfer from the static structure of the reagents to the static structure of the reaction products. In the best cases intermediates were taken into account; however, these were also presented as static structures. It is absolutely common knowledge amongst chemists that molecules, including biopolymer molecules, are subjected to internal motions: the atomic vibrations proceeding at a subpicosecond time scale, fluctuations of a side radical at a pico-nanosecond time scale, conformational rearrangements in the millisecond range, and even slower intramolecular movements. The problem with the molecular dynamics of biopolymers is not limited to simply stating and describing these motions but expands into establishing the role of these dynamic events in the recognition process, catalytic conversions, as well as intra- and extracellular signalling. At the time of writing, the most discussed topic is the role of dynamic factors in enzymatic catalysis [16]. 

The role of dynamics in enzymatic catalysis was first brought under discussion in the induced fit hypothesis formulated by Koshland [17]. According to this hypothesis, no ideal compliance exists initially in the structure of the enzyme active site and the substrate which would enable procession to the execution of chemical conversion immediately after the formation of the enzyme–substrate complex. The process is supposed to be preceded by a conformational adjustment of the complex; i.e., a certain relocation of the atoms, which provides the necessary concordance of the chemical bonds subjected to conversion and a portion of the enzyme active site participating in the catalytic process. 

The concept was confirmed by pre-stationary kinetic data. Such changes may be observed using rapid kinetic methods, such as stopped-flow in the millisecond range and relaxation methods (T-jump) in the microsecond range [18]. As an example, *Fig. 3* shows the curves obtained by the stopped-flow method for the splitting of the base reaction (8-oxoguanine), which is catalyzed by 8-oxoguanine-DNA-glycosidase. The conversion was followed by fluorescence of tryptophan residues. At the first stage, the changes in conformation are clearly visible, whereas when several stages are recorded, the release of the reaction product (8-oxoguanine) is distinctly observed only at the final stage [19]. 

The time range in the use of relaxation methods is significantly expanded by the application of modern lasers capable of irradiating systems via femtosecond impulses, thus generating a T-jump within such a short time period [20, 21]. Moreover, if the solution contains a reagent with p *K*_a _ of the excited state significantly different from that of the ground state present in the solution, a pH jump may be performed via a laser impulse [22, 23].

**Fig. 3:**
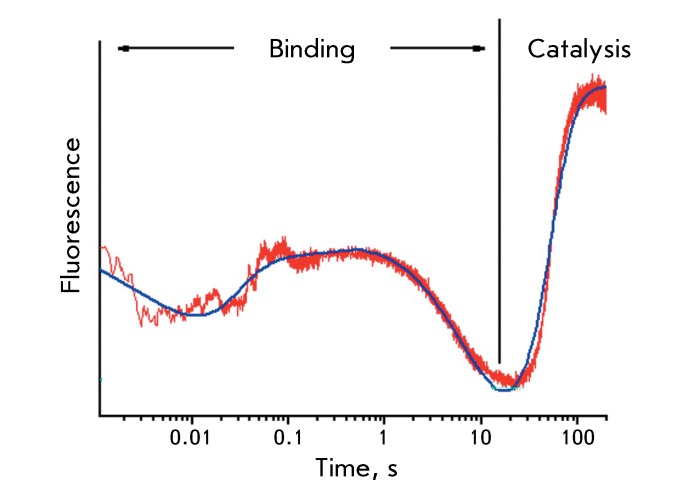
Kinetic curve of the changes in fluorescence intensity (arbitrary units) during the initial phase of the reaction of 8-oxoguanine excision from the oligonucleotide containing an oxidized guanine residue.

The essential dynamics problem is the mechanism by which the system switches from one regime of functioning to another, significantly different, regime. The question already arises with regards to enzymes and enzymatic complexes that possess several catalytic functions manifesting themselves in a certain order. This relates to all the multifunctional enzymes which realize the sequential switching of different functions through a “swinging arm” that is capable of reaching various active sites. A number of such enzymes are known. For example, there is a fatty acid synthase which represents a complex of proteins that catalyze the sequential lengthening of the fatty acid carbon backbone by two-carbon fragments [24]. During the whole process, the growing chain of carbon residue is bound by a thioether bond to the SH-group of phosphopantothein 

-ОРО _2_^-^ -O-СН _2_ -С(СН _3_ ) _2_ -СНОН- -СО-NH-(CH _2_ ) _2_ -CO-NH-(CH _2_ ) _2_ -SH, 

which is covalently bound by a phosphodiester bond to the serine residue of the acyl carrier protein (ACP). The arm contains a large number of σ-bonds and is therefore highly flexible. This allows the acyl residue to move alternately between the active sites catalyzing sequential stages of the biosynthesis of fatty acid from acetyl residues. The primary source of acetyl residues is the acetylated coenzyme A, CoAS-COCH _3_ , the main product of the catabolism of carbohydrates, fats, and a number of amino acids. The acetyl residue is carboxylated, and the malonyl residue formed is transferred from coenzyme A to ACP via the reaction  

СоАS-СОСН _2_ СОО ^-^ + АСРSH → → АСРS-СОСН _2_ СОО ^-^ + СоАSН. 

*Fig. 4* represents a scheme of the processes that occur in all the two-carbon fragments introduced during the process. Malonyl-ACP is the direct donor of these fragments; it binds to the growing chain resulting in the detachment of CO _2_ and the cleavage of the bond of the fragment with the protein, reduction of the fragment to –CHOHCH _3_ , its dehydration to –CH=CHCH _3_ , and reduction to –CH _2_ CH _3_ . 

Clearly, each reaction proceeds with the participation of its own active site. The active sites may reside in different polypeptide chains (in eubacteria) or in one polyfunctional protein (in eukaryotes, including humans). The swinging arm must transport fragments of COCH _2_ R in a definite order to the four active sites for the procession of all sequential conversions. 

The notion of intrinsically unordered proteins is a problem which has recently come to light and requires further study from the perspective of molecular dynamics [25–27]. Currently, there are a large number of such proteins which, in contrast to the commonly held view, function in the absence of a definite tertiary structure. Such proteins are unlikely to exist in the form of a statistically coiled polypeptide chain. In all likelihood, they represent an ensemble of rapidly, mutually transferring conformations in the solution. The predominance of proteins with an unordered conformation is typical of many neurodegenerative diseases, such as the Huntington disease and spinocerebellar ataxia (disorders of the gait and other types of movement coordination). However, many proteins with an unordered structure or at least containing rather expanded (more than 50 amino acid residues) unordered fragments are encountered within the established norm and more often in eukaryotes than in unicellular organisms. Among such proteins, the transcription factors and proteins responsible for chromatin remodeling and intracellular signalling occur more often. This certainly does not mean complete disorder. This can be supported by the fact that many of these proteins become structured after binding to their targets. The absence of order creates a serious problem for the elucidation of their spatial structure, since these proteins do not give rise to reflexes during the X-ray analysis. Meanwhile, more data has been accumulated pertaining to the fact that these unordered parts are most typical of polyfunctional proteins. In all likelihood, the conformations with an affinity to different partners are also present among the conformations of these pseudo-unordered proteins.These proteins are typically characterized by a small content of bulky hydrophobic amino acid residues and an increased content of polar and charged residues. 

At the time of writing, the theoretical investigation of biopolymer molecular dynamics is limited by the capabilities of computer techniques. The calculation of molecular dynamics assumes that a stepwise procedure is used, and it requires femtosecond time increments. Even for the modern supercomputers and software, the advance for several tens of nanosecond incrementscan only be attained for the biopolymers consisting of thousands atoms. Meanwhile, the most interesting conformational events occur in the micro- and even millisecond ranges.  

**Fig. 4:**
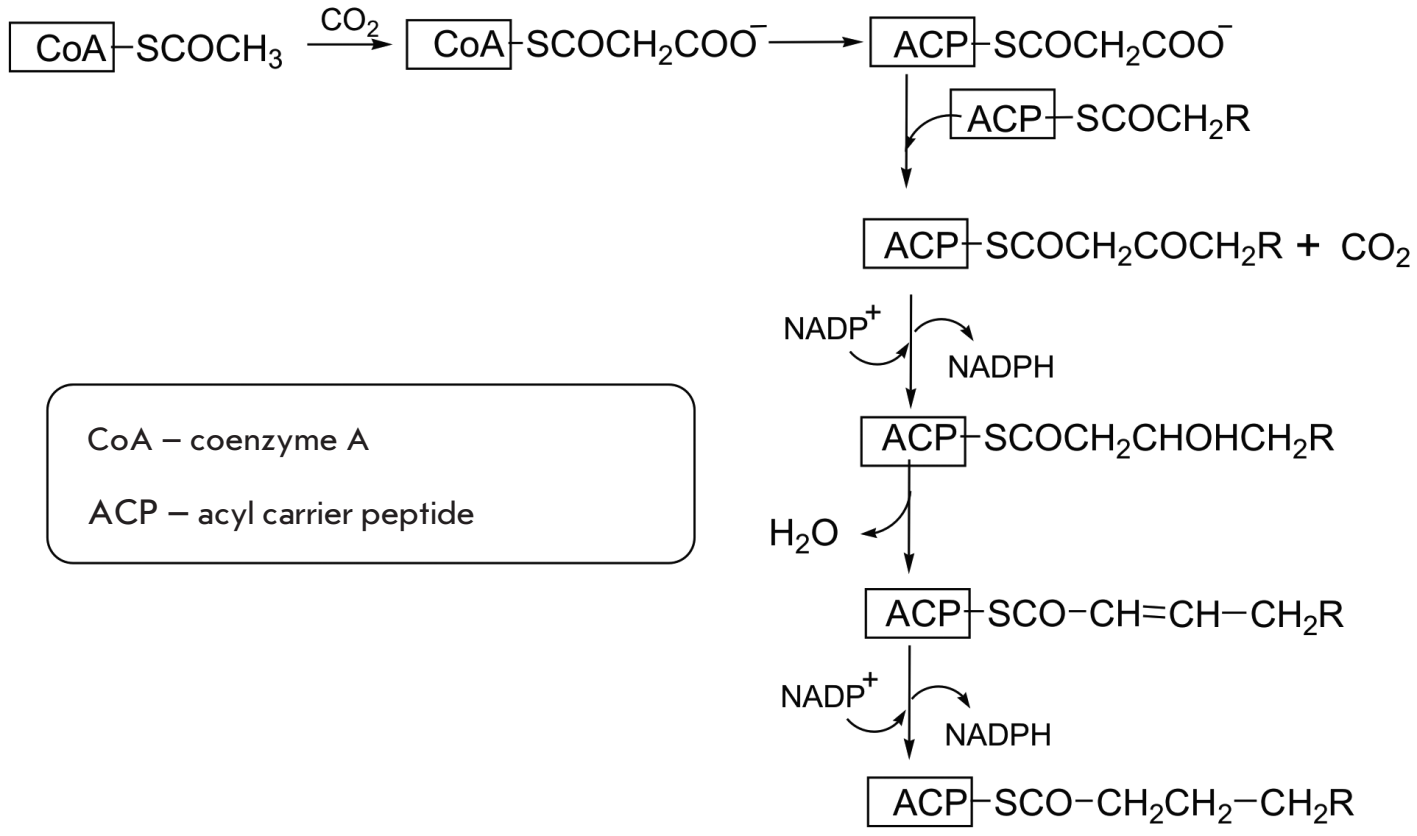
Scheme representing the lengthening of two-carbon fragments catalyzed by fat acid synthase.

Most studies focused on the physicochemical ground of the vital activity, in particular in the case of quantitative characteristics, were carried out *in vitro* . Most of the values obtained may be to a significant extent related to intracellular processes, especially to those in eukaryotic cells. A common eukaryotic cell may carrya large number of biopolymer molecules. The conditions in its cytosol do not significantly differ from those in a test tube.This may be demonstrated via a simple calculation for a spherical cell with a linear dimension of 20µm, which is typical of a eukaryotic cell. A spherical cell of a 20-µm diameter was used to simplify the evaluation. For illustrative purposes, it is more convenient to perform calculations in Daltons (Da) as mass units and angstroms as length units (they can be qualified as the “cell” ones). Since 1g = 6 × 10 ^23^ Da and 1cm = 10 ^8^ Å, the density is measured in 1g/cm ^3^ = 0.6Da/Å ^3^ . The cell volume amounts approximately to 4 × 10 ^15^ ; the volume of a relatively large protein molecule (approximately 100kDa) is ~ 10 ^5^ Å ^3^ . Assuming that the proteins occupy 10% of the cytosolic volume, 4 billions of such molecules can be accommodated in one cell. Therefore,it can be reckoned that cytosol conditions (with allowance made for the increased viscosity of the 10% protein solution) do not differ significantly from those in a tube. The arrangement of proteins on the cell surface can be estimated in a similar manner. Assuming that 10% of the plasma membrane surface is occupied by embedded proteins (functioning as receptors, transport and channel-forming proteins, etc.), it is simple to calculate that ~4·10 ^5^ proteins of 100 kDa can be accommodated therein. 

Both *in vitro* and whole-cell studies provide data on the physicochemical characteristics of biochemical processes averaged on the entire set of the molecules involved in it. Therefore, the new possibilities that open up with the development of techniques for dealing with single molecules represent a new and important direction of research. On one hand, these investigations are aimed at elaborating methods for the sequencing of single DNA molecules; a considerable degree of progress has been made in such work in recent times [28]. The second direction is the investigation of reactions catalyzed by a single enzyme molecule. In this case, the reaction should be accompanied by a fluorescence change. Cholesterol oxidase (EC 1.1.3.6) [29], which catalyses cholesterol oxidation through molecular oxygen, can be used as an example.The reaction involves two stages: 

**Fig. 5:**
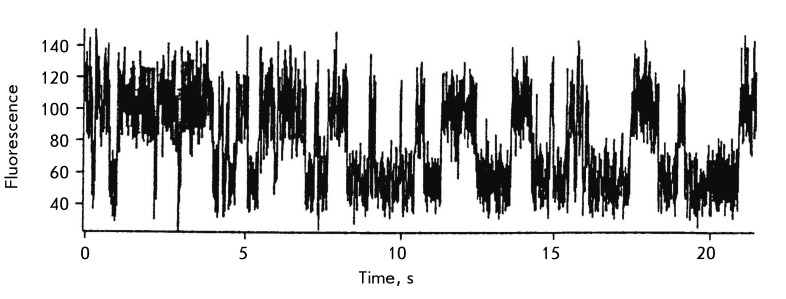
Registration of cofactor fluorescence during catalytic oxidation of cholesterol.

Cholesterol + FAD ↔  ↔ cholesterol oxidized + FADH _2_

FADH _2_ + O _2_ ↔ FAD. 

Cholesterol is oxidized by fluorescent cofactor flavin adenine dinucleotide (FAD) bound to the protein moiety of the enzyme. During the process of cholesterol oxidation, FAD is transformed into the non-fluorescent reduced form FADH _2_ . At the second stage of the reaction, FADH _2_ is oxidized by molecular oxygen to the initial FAD. Each separate catalytic process is characterized by the attenuation and intensification of fluorescence, allowing one to follow each process of enzyme functioning. *Fig. 5* shows the results of the registration of fluorescence upon the catalytic oxidation of cholesterol. 

The investigation of macromolecule folding is another important aspect of the application of single molecule spectroscopy. Thus, single molecule fluorescence can be used to observe the dynamics of the formation of the spatial structure of RNA, which can also be recorded via the FRET (Forster resonance energy transfer) technique [30]. The intensity of the fluorescence energy transfer between the fluorophores bound to certain points of a fluorescence donor being irradiated and its acceptor is in inverse proportion to the sixth power of the distance between them. Any changes in the distance between the fluorophores during the formation of the spatial structure affect the fluorescence of an acceptor between them. The acceptor fluorescence will change with changes in the spatial structure. 

The problems considered above, which arise in physicochemical biology, are related to proteins and nucleic acids, the investigation of which was a priority in research in the 20th century. When discussing new horizons in physicochemical biology, one should mention the demand for increasing the level of attention paid to other groups of compounds, with reference primarily directed at carbohydrates of an irregular structure, which play a significant role in the provision of a number of highly selective processes (i.e., the distribution of biochemical processes between cellular organelles). In addition to their cognitive significance, these directions will contribute significantly to the design of new drugs, the investigation of their interactions with living organisms, as well as their transformations and side effects. Therefore, these directions have the potential of becoming important elements of medicine in the 21st century.  
